# Integrating Social Emotional Learning Strategies in Higher Education

**DOI:** 10.3390/ejihpe10030061

**Published:** 2020-08-26

**Authors:** Chiara Elmi

**Affiliations:** Department of Geology and Environmental Sciences, James Madison University, 801 Carrier Drive MSC 6903 Room 3232, Harrisonburg, VA 22807, USA; elmicx@jmu.edu

**Keywords:** higher education, social and emotional learning, science learning

## Abstract

Social and emotional learning (SEL) strategies develop skills linked to cognitive development, encourage student focus and motivation, improve relationships between students and teachers, and increase student confidence and success. More attention should be paid to students’ emotions in higher education to enhance students’ engagement in the classroom and improve social awareness (i.e., respecting others, understanding other perspectives, providing help to those who need it), motivation, and academic achievement. This article focuses on the implementation of practices that promote SEL in higher education and science, technology, engineering, and mathematics (STEM) programs. The paper aims to assess the academic and behavioral-related outcomes of applying SEL in mineralogy, an Earth science introductory course in a four-year university. The results of the present paper reveal that instructional practices supporting SEL are suited for engaging and stimulating learners’ multiple intelligences. The observed student course assessment performance suggests that integrating SEL may be a viable strategy for promoting student interest in science, building stress resilience, and creating more positive engagement with students. The instructional practices reported in this paper could support science instructors in designing teaching methods that promote self-management and social awareness to increase students’ academic outcomes.

## 1. Introduction

Students with diverse abilities comprise a growing population on college and university campuses. With increases in students from diverse backgrounds [1] and students with disabilities [2], the university classroom is becoming more diverse. Adult students used to be called nontraditional students. They have become common across U.S. higher education, particularly in metropolitan universities and colleges that serve urban regions with large populations of place-bound working adults in need of higher education and the certifications that come with that education. Adult students in higher education in the United States (U.S.) are as diverse as the over 4000 colleges and universities that they attend [3,4]. With equal-access requirements and increasing rates of enrollment of students with disabilities and adult students in higher education, universities must find appropriate and efficient ways to create accessible materials that benefit and support all students [5]. Because each student is unique, teachers must use diverse strategies suited to students’ broad array of abilities and multiple intelligences, enabling all students to be true participants in a community of learners [6,7].

U.S. education policies over more than a decade have focused on labeling schools and students on the basis of standardized test results, creating a focus that has increasingly ignored the affective and social needs of scholars as well as their engagement in empowering forms of learning [8]. Durlak et al. [9] found that among 148,000 students, only 29% indicated that their schools provided a caring, encouraging environment. Instructors can serve as key players in ensuring accessible education for all students by building supportive courses that foster student engagement. Inclusive teaching orchestrates the learner’s experience so that all aspects of brain operation are addressed (e.g., thoughts, emotions, imagination, and predispositions) [10]. Brain research has provided educators with a better understanding of instructional practices that not only are essential for students with special needs but also benefit their peers [7]. Anderson et al. [11] described good teachers as people who care about their discipline, care about teaching, and care about students, powerfully influencing students’ engagement with the subject matter, enthusiasm for learning, and aspirations for the future. These authors argue for the need to recognize teaching in higher education as cognitive, emotional, and embodied work.

Education is one of the largest application areas for the construct of emotional intelligence (EI). Emotional intelligence refers to a set of hierarchically organized core competencies and skills for identifying, expressing, processing, and regulating emotions—both in oneself and others [12]. Emotional intelligence is an intelligence dimension that has a significant impact on various life outcomes, such as life satisfaction and job performance [13]. Higher levels of emotional intelligence are associated with a variety of general positive intrapersonal outcomes. These outcomes include greater subjective well-being assessed through indices such as positive affect, life satisfaction, and better mental health [14]. 

The Collaborative for Academic, Social, and Emotional Learning (CASEL) defined social and emotional learning (SEL) more than two decades ago. SEL is the process by which children, adolescents, and adults acquire and apply the necessary knowledge and skills to understand and manage emotions, set goals, show empathy for others, establish positive relationships, and make responsible decisions [15,16]. The definition of social and emotional learning has been recently expanded to include an equity lens, which states that, “Through strengthening our SEL competencies we are better able to connect across race, class, gender identity, sexual orientation, learning needs, and age” [17]. 

The emphasis on social and emotional learning is rapidly growing at all levels of the education delivery system and in professional and continuing education programs [18,19]. SEL is an approach; it is integral to quality teaching and learning [17,20]. An effective SEL approach is designed to improve the quality of classroom interactions, academic development, motivation to learn, and teacher–student engagement through empirical practices, classroom activities that infuse social–emotional competencies into teacher–student interactions [16,20]. Looking at everything from a student’s overall wellness to what drives student interactions, teachers are given the diagnostic tools to look beyond content delivery [20]. Research in higher education populations demonstrates that social and emotional adjustment is associated with positive academic outcomes, including academic performance and retention. Furthermore, social and emotional skills extend beyond academic contexts and outcomes, such as success in work, positive interpersonal relationships, and better mental health and overall well-being. Thus, the value of SEL is vital in higher education [18].

Social and emotional learning concerns the development of emotional intelligence (EI) skills, including self-awareness, self-management, social awareness, relationship skills, and responsible decision-making [15,21]. Research has demonstrated that social and emotional competences in these five SEL domains are critical to higher education students’ development, adjustment, and success [18]. Many districts and several states have created developmentally appropriate SEL standards for middle and high school programs using the CASEL framework as a guide. While there is a growing interest in the SEL approach, nevertheless, social and emotional competences in higher education have not yet been as organized, structured, or uniformly applied as those for younger scholars [18]. The goal of this paper is to shed light on the effectiveness of implementing SEL strategies in higher education, especially in science, technology, engineering, and mathematics (STEM) programs. The paper focuses on the academic and behavioral-related outcomes of applying SEL in mineralogy, an Earth science introductory course in a four-year university.

## 2. Design and Methods 

### 2.1. Course Design

Mineralogy is a fundamental introductory course in a four-year university that prepares students and exposes them to the content and theory that define their academic major. Previous knowledge in high school chemistry and physical geology is strongly recommended to enroll in the course. The instructional approaches described in this paper were applied in two semesters on a pool of approximately 45 students at a comprehensive public higher education institution in the United States of America. The class met two times a week, with each lecture lasting 75 min, and each laboratory meeting lasted 2 h. Laboratory activities were performed soon after the lecture in order to reinforce students’ understanding of the lecture topics and long-term retention of the information that they learned. 

Students self-selected into the course via enrollment. Students varied in academic rank and age, with the majority of adult students being in their sophomore and junior years enrolled in engineering, physics, and Earth and environmental science subjects. Of the average students enrolled, 45% were adult (nontraditional), 32% transferred from another postsecondary institution, and 23% were attending a postsecondary institution for the first time.

The course was designed focusing on the self-awareness, social awareness, and relationship skills SEL competencies. Learning goals and objectives were a central element of the syllabus and the course in order to enable each student to achieve the best knowledge outcome. Table 1 shows the SEL skills targeted in interventions in relation to core SEL competency domains in the specific class considered in this paper. 

### 2.2. Pedagogical Approach

Mineralogy and crystallography are among mathematics, physics, and chemistry, the oldest branches of science. Mineralogy is established both as an independent science and as a support discipline for many other branches of science, such as solid-state physics, chemistry, and materials science. A social–emotional learning approach provides flexibility in instructional presentation and student engagement [22]. As mineralogy is traditionally considered a challenging field, the enhancement of a SEL approach can aid in stimulating interest and motivation for learning science and building stress resilience. 

The SEL skills prioritized for assessment and instruction were self-management, social awareness, and relationship skills. Kolb’s theory of the experiential learning cycle [23] was also considered to increase student achievement and to meet the wide variety of learning needs and abilities in the college classroom.

The main goal of the course was to give students the opportunity to develop the set of skills necessary to understand mineral-forming processes as well as recognize potential uses of minerals as raw materials while accommodating a wide range of individual preferences and abilities. In the first part of the course, students were introduced to the basic principles behind the arrangement of atoms to form crystal structures; how these atoms are coordinated and bonded; and how this is reflected in the external form, chemical composition, and physical properties of inorganic compounds. Symmetry is a challenging concept for students to master because it is an abstract notion. In mineralogy and chemistry, the interaction of symmetry elements on atoms determines crystal structures, and the systematic repetition of atoms in space is a reflection of the crystal forms exhibited in hand samples.

Students may have a difficult time mastering concepts of chemistry and mineralogy because it is typically their first formal encounter with 3D visualization, and the crystal structures may appear to be abstract and, thus, overwhelming for many students. To minimize frustrations, several options were provided for accessing the information on the topic, such as photos and images that represent symmetric objects to explain the geometrical arrangement of atoms forming crystal structures, software to manipulate and visualize complex 3D crystal shapes, and wood models that represent common crystal shapes in nature.

Hands-on activities using wood crystal models and crystal structure modeling software, such as CrystalMaker [24] and KrystalShaper [25], allowed students to gain a tactile and visual, i.e., 3D, physical representation of an abstract concept such as the internal structure of a compound. The use of Escher tessellations [26] and examples of symmetric objects in everyday life became an effective way to convey the abstract concept of symmetry in minerals using notions with which students were already familiar. These specific exercises were supported by other, more traditional means of representation (e.g., PowerPoint presentations, videos, online resources, and textbooks). These activities and assignments maximized students’ self-awareness on the material and stimulated their curiosity. 

In the second part of the course, students learned how to identify the most common minerals forming rocks in hand specimens using optical microscopy (they identified the common rock-forming minerals in thin sections) and X-ray diffraction. Several options were provided for accessing information, such as hand specimens of minerals, online resources to recognize minerals via petrographic microscope, and real-world research activities using the laboratory equipment to recognize minerals from real analytical data.

Students’ overall performance was measured via three summative midterm exams. Each exam included essay questions or open-ended questions that involved high levels of Bloom’s taxonomy (analysis, synthesis, and evaluation). The exams were divided into two parts: one part tested the fundamental basic concepts via essay or open-ended questions, and a second part was devoted to the comprehension of laboratory activities using real-world exercises (e.g., interpretation of the X-ray pattern of an unknown phase, identification of hand specimens, and recognition of common rock-forming minerals in thin sections).

### 2.3. Data Collection and Analysis

The present study was focused on the education instructional experiences of the instructor over the course of two years. The research aimed to assess the quality of class activities integrating SEL strategies. The hypothesis question was as follows: as mineralogy is traditionally considered a challenging field, can the enhancement of an SEL approach aid in stimulating interest and motivation for learning science, building stress resilience and positive engagement with students?

Students were observed during the laboratory activities throughout the semester, and the core SEL competency domains were assessed using a rubric (Table 1) designed by the instructor following the Collaborative for Academic, Social, and Emotional Learning (CASEL) framework. The rubric was created to help students understand what self-awareness, social awareness, and relationship skills entail. All identifying information has been removed, and there is no way that it could be linked back to the subjects from whom it was originally collected (through a key to a coding system or by any other means).

## 3. Results and Discussion

Willis [7] observed that as students experience the connection between practice and progress in achieving their goals, they will appreciate their teachers for having provided them with the keys that unlock the doors to their aspirations. Instructional strategies that include brain-friendly strategies such as social and emotional learning (SEL) make the classroom a supportive and welcoming place where all students experience the joy of learning and contribute to enhancing students’ performance. Moreover, SEL helps to reduce teacher stress and create more positive engagement with students [27]. SEL strategies provided students the tools to overcome obstacles and learn to their fullest potential. By using SEL, students were encouraged to be effective learners developing self-awareness, social-awareness, and relationship skills. 

The design of a positive, inclusionary learning environment can contribute to minimizing stress related to the assimilation of complex concepts and new terminology. Accomplished teachers base their practice on the fundamental belief that all students can learn and meet high expectations. Acknowledging the distinctive traits and talents of each learner, teachers are dedicated to and skilled at making knowledge accessible to all students. Educators are thus passionate about building meaningful relationships with students who can advance their understanding and experience success. The pedagogical approach described in this paper can contribute to making teaching and learning processes more fruitful and rewarding. Planning SEL goals as reported in Table 1 was time-consuming, but efforts were rewarded by students’ improved self-confidence, social awareness (i.e., respect others, understand other perspectives, provide help to those who need it), motivation, and academic achievement. The main achievement was to demonstrate that success is measured not only by tests and grades but also by the ability to practice the executive functions of planning, time management, and prioritization (e.g., set plans and work toward goals). Attendance and participation during class averaged nearly 94% in the first year and 97% in the second year. These data were compiled from the number of exercises that were completed and handed in during class meetings. 

SEL led to improved student behavioral skills. Peer support mediates between relationship skills and academic achievements. At the end of the course, students discovered that they could not succeed and grades could drop if they did not work collaboratively with their classmates and instructor during class meetings. Homework and in-class activities honed their comprehension of the course concepts, reducing stress to succeed in the course. The real-world activities (e.g., identification of common minerals in hand samples, observation of symmetry elements using art and everyday objects, etc.) aimed to urge students to realize their potential and reinforce their critical thinking and problem-solving skills. The goal-setting process leads to strong collaboration among students.

In the course evaluation survey at the end of the course, specific questions were added to the evaluation questionnaire asking about the impact of the teaching methods being used. To the statement, “the use of essay questions in course assignments and in the exams increased the amount that I learned in this course”, 91% of the students indicated that they agreed. To the question, “In general, are you satisfied with the activities proposed by your instructor?”, 95% of students recognized that the teaching approaches used were positive factors and acknowledged that they had benefited from the pedagogy applied. Written comments by students in the summative evaluations supported this assessment. Although there were a few negative comments about the design of the course, the prevailing attitudes are summarized in the following extracts:

“Having lab time and being able to converse with other students helped me to learn the material much easier.”

“This was a very fun class, I learned a lot.”

“The hands on experience was extremely valuable for understanding complex concepts and information related to crystal structure, mineral identification and composition and other topics covered in the course.”

“I found the homework assignments and the lectures very helpful in learning the material and studying for the tests.”

“Professor used pedagogical methods and academic tasks that required students to learn actively, responsibly and cooperatively.”

“I think the lecture slides and homework assignments were extremely helpful and I think the instructor enthusiasm and knowledge on the subject really helped me learn and take more out of this course.”

“This course is a lot easier once you understand the material rather than attempting to memorize it.”

### 3.1. SEL Strategies to Improve Self-Awareness

Self-awareness includes the ability to accurately assess one’s strengths and weaknesses. This knowledge enables people to assess which endeavors are within their abilities and what skills they need to develop. Self-awareness is the foundation for good self-management. Self-management concerns regulation of our emotions and behavior and the ability to set goals and pursue them. Self-management depends upon what psychologists refer to as executive functions, the skills needed to focus our actions in pursuit of an objective despite distractions: response inhibition, interference control, working memory, and cognitive flexibility [21].

In the first week of the course, it was elucidated to students why writing was integrated into their learning process. Specifically, it was explained to them that (i) writing helps them to synthesize and articulate complex concepts, (ii) learning how to write in science can be beneficial when they will be required to write a report, and (iii) writing can support students in their progress to accomplish mastery. Review questions in the form of essays were assigned as homework to practice metacognition, improve self-awareness on the comprehension of concepts, and summarize the main key points outlined in the textbook.

Inspecting mistakes opens up a space for enhancing students’ conceptual understanding [28]. Mistakes were valued as learning opportunities and allowed students to build self-confidence along with knowledge. The feedback on student work was provided by a week after its submission. The prompt and explicit feedback included suggestions for improving future assignments and the corrections of conceptual mistakes; thus, students could learn from them. These interventions helped students to keep track of their preparation, accurately judge their own performance, and assess their strengths and limitations.

Review sessions (evaluation of homework and tests) of 10–15 min helped to define strategies that were either incorrect or unjustifiable, improve the quality of assignment submissions, and develop self-study practices and test preparation approaches that allowed students to retain information and connect it with future learning. Review questions in the form of essays educated students to build confidence in their writing and self-study abilities, improve their reading comprehension skills, and enhance their ability to restate key information they have read in the textbook. Although the evaluation of the writing assignments was time-consuming, the additional work was outweighed by an enhanced educational experience for students.

Students had the opportunity to attend a seminar on CO_2_ sequestration and the interaction with clay minerals. One week before the seminar, students were lectured on clay minerals and their applications. Students were divided into groups, and a leader was assigned. The group assignment consisted of preparing and presenting a poster to classmates on different themes related to carbon storage and the interaction with clays. Students had the opportunity to discuss their group theme with the seminar speaker before the talk. Students worked collaboratively for a week to prepare the poster, and then they presented their findings to their peers. This team activity encouraged students to be the agents of their own learning and promoted communication, social, and emotional competencies such as social awareness and relationship skills. 

### 3.2. Fostering Collaboration in the Classroom to Improve Social Awareness and Relationship Skills 

Inclusive courses, when successfully planned and taught, become places where social interactions are founded on the appreciation of similarities and differences [7,29]. In the sense of inclusive education, it needs to be clear that all students are welcomed, appreciated, and valued members of the classroom, no matter what their differences may be [29]. The learning environment needs to provide stability and familiarity [10]. Social awareness is the foundation for relationship skills. We need to know what others need and want and how they are likely to respond to what others do to know how we should approach them [21]. The teaching strategies reported in this paper can be suited for engaging and stimulating all learners. Since the beginning of the semester, students were encouraged to help and teach one another during the in-class activities, establishing a sense of community and classroom norms that assumed that all people needed help. 

Schutte and Loi [14] observed that higher levels of emotional intelligence are generally associated with a variety of interpersonal outcomes, including more cooperative behavior. Bellocchi [30] showed that the social actions of numerous students and the teacher led to the construction of emotion management and how this impacted social bonds. Several authors mention the importance of fostering collaboration through the use of peer tutoring investigations. Peer tutoring is an effective way to help students to understand the work that they have missed or that they did not understand during the last lesson, but it is not efficient when it replaces explicit teaching by the teacher [29]. Classwide peer tutoring (CWPT) is highlighted as a method of supporting inclusive education [29]. In the course considered here, two students (tutors) who understood the material were paired with a student who was struggling and/or a student who missed a lecture (tutee). The tutor–tutee role was usually rotated because it gave each student the opportunity to benefit from teaching the other student. With the CWPT approach, students become producers and consumers of education [29]. 

Hands-on laboratory activities using wood crystal models, Escher tessellations [26], and crystal structure modeling software CrystalMaker [24] promoted peer collaboration and independence. The CWPT strategy encourages students to foster collaboration to problem-solve and contributes to limiting frustrations on the resolution of challenging tasks [31]. The objective of peer collaboration is to provide experiences and develop student goals based on the individualized realistic challenge, which connects students to knowledge by communicating to them high expectations while confirming that they have the capacity to reach these goals [7]. However, challenges should not exceed students’ level of capability; otherwise, exasperation replaces motivation, reduces engagement, and generates stress. 

With the application of the classwide peer tutoring strategy, over time, the joy of learning replaced frustration and avoidance of challenges. The peer collaboration process described in this paper allowed the instructor to have time to work with a few students who needed more assistance, whether remedial or advanced. This classroom arrangement became more effective as it became routine at the end of the semester. Students improved their academic and communication skills, gained a better understanding and acceptance of diversity, and improved their social awareness and relationship skills.

### 3.3. Engaging Students Using SEL

Engagement involves stimulating students’ interest and motivation to learn through creative, hands-on, and meaningful instruction. Neurological research confirms the value of connecting students to academic material by engaging their interests, fostering positive emotions, and making real-world connections [7,10]. Because of the increased external stressors in today’s society, it is crucial that instructors build supportive classroom communities in which students retain (or regain) the excitement they felt when they entered kindergarten [7]. 

Learning is a natural response to encountering something new. The assimilation of knowledge occurs when a learner encounters a new idea, and that idea must be linked to that with which students are already familiar. When students reach or exceed their goals, they set new short- and long-term goals. Learning is as natural as breathing, but it can be either inhibited or facilitated. Stress and threats affect the brain differently from peace, challenges, boredom, happiness, and contentment. Stress management and relaxation should be fully incorporated into the learning process [10]. If high stress or negative emotions have overloaded the amygdala, the affective filter will block the passage of data into the memory. On the other hand, pleasurable, positively reinforcing, and intrinsically motivating stimuli unlock the gates of the limbic system to facilitate active information processing [7,32]. 

Processing information requires time. Neuroimaging confirms that the least efficient memory strategy is routinized memorization of facts and skills taught in isolation [32]. 

In addition to promoting academic success, writing assignments and hands-on activities developed self-awareness and self-management (i.e., attention to detail, reflection, and perceptiveness). Creating or mimicking real-world experiences through demonstrations, projects, or student-centered discussions instills personal relevance into academic content areas and shows students why the information is, or could be, personally important to them. If students cannot relate to the new material, their neural networks will be less able to process it and retain it in the long-term memory, leading to frustration and discouraging classroom experiences [33]. The use of Escher tessellations [26] and examples of symmetric objects in everyday life shows that when lessons are adapted for multiple intelligences, the content is more likely to be personally meaningful to students and to connect to their relational memories of successful patterning and long-term retention [7]. 

The recognition of hand specimens of common minerals was conducted as an investigation. This in-class assignment included short-answer questions that improved students’ comprehension of the nature of scientific evidence and how a researcher thinks and solves problems. Students were asked to create a mineral identity card that contained physical properties, economic significance, and fun facts related to mineral resources. The search for the uses of and facts related to the mineral samples that students observed in class facilitated processing a substantial amount of novel information and retaining it in the long term.

### 3.4. Limitations and Future Directions

The present study offers a platform for more in-depth exploration of implementing SEL across different cohorts and STEM disciplines. Because there is no standardized approach in measuring SEL skills, the present theory-driven research aids in the assessment of successful educational practices that effectively promote SEL among college students majoring in STEM disciplines. However, some limitations should be considered. Evidence of the effectiveness of SEL strategies in higher education is limited. The limitations of the study include a small sample size. More data across multiple outcome areas are needed. Further follow-up investigations should be conducted to confirm the long-term impact of SEL competences. Future in-depth quantitative and qualitative evaluations of the implementation of SEL in other similar courses are warranted.

## 4. Conclusions

This study focused on the experiences of the instructor over the course of two years. The reported outcomes and practice form the basis for discussing the implications of social–emotional learning in higher education settings. Moreover, the results of the present study reveal how important it is to support students in developing a sense of community and motivation and fostering academic success through emotional intelligence. A positive attitude, a proactive approach to life, a tendency to set goals, perseverance, effective support systems, and empathy are effective factors that can contribute to helping students to become more confident learners. Helping students to develop skills that promote their social and emotional well-being seems to be a long-term investment in higher education, as it is in earlier educational settings.

Brain research has considerable implications for education because it addresses the basic concepts upon which teaching and learning are based. Teachers need to understand that students’ feelings and attitudes are always involved and determine the learning process.

Because it is impossible to isolate the cognitive from the affective domain, the learning environment needs to be supportive and marked by mutual respect and acceptance both within and beyond the classroom [10]. Emotions shape the landscape of our mental and social lives. Like geological upheavals in a landscape, they mark our lives as uneven, uncertain, and prone to reversal [34]. Hasson [35] observed that emotions give feelings of self-respect and self-esteem. Emotional intelligence creates the unique opportunity for students to discover their potential and foster empathy by breaking down barriers.

Durlak et al. [9] show that students exposed to social and emotional learning (SEL) in school continue to do better than their peers on a number of indicators: positive social behaviors and attitudes, skills such as empathy and teamwork, and academics. Moreover, Belfield et al. [36] reviewed the available evidence on the economic value of SEL. These authors found that the measurable benefits of SEL exceed the costs, often by considerable amounts (on average, for every $1 invested in SEL programming, there is a return of $11). By understanding people’s emotions, educators can help students develop their full potential as lifelong learners.

The Nobel laureate Paul Romer said, “You do the thing you’re good at. Other people do something else that they are good at. The net effect is better for everybody” [37]. This quote emphasizes how fostering collaboration is an advantage to promoting a social and emotional learning environment. Teacher preparation programs need to focus more on collaborative practices with an emphasis on differentiated instruction and accessible options. Researchers are learning that for inclusive education to be meaningful and effective, peer-to-peer relationships are more than an outcome; they are a critical component of the process of inclusive education [29,31,38].

The outcome of the instructional practices described here suggests that mastering the concepts requires time and effort. Promoting student interdependence and independence through student-centered activities, discovery, and hands-on learning experiences enables them to process information by connecting it with prior knowledge and experience, identifying rules, making the science relevant and meaningful, and generating abstract principles. Moreover, fostering emotional intelligence increases empathy, creativity, and a sense of community. The development of awareness of others needs to limit the establishment of stressful situations that can disturb the process of teaching and learning.

Social–emotional learning strategies can be applied to create a powerful and integrated model of human intelligence and learning—a model that respects and celebrates diversity and provides instructors with the tools to meet high standards.

## Figures and Tables

**Table 1:** Skills targeted in interventions in relation to core social and emotional learning (SEL) competency domains. Adapted from Conley [18].

	SEL Competency Domains		
	Self-Awareness	Social Awareness	Relationship Skills
	Recognizing strengths	Identifying appropriate social resources and supports	Communicating clearly and effectively
	Self-confidence	Respect for others	Teamwork
**Anchor standards**	Recognizing filters that impair good communication	Listening with understanding	Relationship problem-solving
	Identifying when help is needed and who can provide it		Conflict management and resolution
			Seeking and providing help when needed
